# Clinical Features of Familial Hypercholesterolemia in Children and Adults in EAS-FHSC Regional Center for Rare Diseases in Poland

**DOI:** 10.3390/jcm10194302

**Published:** 2021-09-22

**Authors:** Joanna Lewek, Agnieszka Konopka, Ewa Starostecka, Peter E. Penson, Marek Maciejewski, Maciej Banach

**Affiliations:** 1 Department of Preventive Cardiology and Lipidology, Chair of Nephrology and Hypertension, Medical University of Lodz, 93-338 Lodz, Poland; 2 Department of Cardiology and Adult Congenital Heart Diseases, Polish Mother’s Memorial Hospital Research Institute (PMMHRI), 93-338 Lodz, Poland; marek.maciejewski@iczmp.edu.pl; 3 Regional Rare Disease Centre, Polish Mother’s Memorial Hospital Research Institute (PMMHRI), 93-338 Lodz, Poland; agnieszka.konopka@iczmp.edu.pl (A.K.); ewastarostecka@wp.pl (E.S.); 4 School of Pharmacy and Biomolecular Sciences, Liverpool John Moores University, Liverpool L3 3AF, UK; P.Penson@ljmu.ac.uk; 5 Liverpool Centre for Cardiovascular Science, Liverpool L7 8TX, UK; 6 Cardiovascular Research Centre, University of Zielona Gora, 65-046 Zielona Gora, Poland

**Keywords:** familial hypercholesterolemia, registry, lipid-lowering therapy, statins

## Abstract

Background: Familial hypercholesterolemia (FH) is a genetic autosomal co-dominant metabolic disorder leading to elevated circulating concentrations of low-density lipoprotein cholesterol (LDL-C). Early development of atherosclerotic cardiovascular disease (ASCVD) is common in affected patients. We aimed to evaluate the characteristics and differences in the diagnosis and therapy of FH children and adults. *Methods*: All consecutive patients who were diagnosed with FH, both phenotypically and with genetic tests, were included in this analysis. All patients are a part of the European Atherosclerosis Society FH-Study Collaboration (FHSC) regional center for rare diseases at the Polish Mother’s Memorial Hospital Research Institute (PMMHRI) in Lodz, Poland. *Results*: Of 103 patients with FH, there were 16 children (15.5%) at mean age of 9 ± 3 years and 87 adults aged 41 ± 16; 59% were female. Children presented higher mean levels of total cholesterol, LDL-C, and high-density lipoprotein cholesterol (HDL-C) measured at the baseline visit (TC 313 vs. 259 mg/dL (8.0 vs. 6.6 mmol/L), *p* = 0.04; LDL 247 vs. 192 mg/dL (6.3 vs. 4.9 mmol/L), *p* = 0.02, HDL 53 vs. 48 mg/dL (1.3 vs. 1.2 mmol/L), *p* = 0.009). Overall, 70% of adult patients and 56% of children were prescribed statins (rosuvastatin or atorvastatin) on admission. Combination therapy (dual or triple) was administered for 24% of adult patients. Furthermore, 13.6% of adult patients and 19% of children reported side effects of statin therapy; most of them complained of muscle pain. Only 50% of adult patients on combination therapy achieved their treatment goals. None of children achieved the treatment goal. Conclusions: Despite a younger age of FH diagnosis, children presented with higher mean levels of LDL-C than adults. There are still urgent unmet needs concerning effective lipid-lowering therapy in FH patients, especially the need for greater use of combination therapy, which may allow LDL-C targets to be met in most of the patients.

## 1. Introduction

Familial hypercholesterolemia (FH) is a genetic autosomal co-dominant metabolic disorder caused by defective clearance of low-density lipoprotein cholesterol (LDL-C), leading to lifelong elevations in circulating concentrations [1]. FH occurs in between 1:250 and 1:300 people, meaning that in Europe there are as many as 3 million patients, and over 64 million worldwide [1,2,3,4]. Several tools can be used for FH diagnosis, including: MedPed, Simon Broome, newly proposed The Familial Hypercholesterolemia Case Identification Tool (FAMCAT), and the most widely used Dutch Lipid Clinic Network (DLCN), which assess the clinical probability of a FH diagnosis [2]. Molecular diagnostics testing is recommended (but phenotypic diagnosis is sufficient to start therapy) and allows for genetic testing for mutations in genes responsible for FH: the most common are in the LDL-C receptor (LDLR), apolipoprotein B (apoB), proprotein convertase subtilisin kexin type 9 (PCSK9), and more rarely the LDLR adaptor protein (LDLRAP1) and apolipoprotein E (APOE) [1,5,6]. In most individuals the mutation is present in one copy of gene with a heterozygous state. Homozygous FH is more severe, with higher levels of LDL-C and poorer prognosis, even at a young age [7]. Homozygous FH affects a relatively small population of patients, with a prevalence of 1:160,000 to 1:300,000 [7].

It is estimated that FH affects approximately 150,000 people in Poland [8,9]. However, most of these patients are still unaware of their condition, and currently only about 5% of FH patients in Poland have been diagnosed. Underdiagnosis is a systemic problem in many European countries that needs to be immediately resolved as FH leads to early cardiovascular mortality and morbidity due to the lifetime exposure to high levels of LDL-C [6,10,11]. Early development of atherosclerotic cardiovascular disease (ASCVD) is common in FH patients [6,10,11]. However, prospective studies show that other risk factors such as hypertension, diabetes, and smoking are also of utmost importance [6]. It has been shown that, even in patients with the comparable level of LDL-C and the same genetic mutation, the risk of adverse events may significantly differ [6,12]. Nevertheless, the overall cardiovascular risk in patients with FH is high, even when there are no other risk factors. Risk in these individuals is classified as high to very high according to European Society of Cardiology/European Atherosclerosis Society (ESC/EAS) guidelines [13], and to extremely high according to recent statements made by the International Lipid Expert Panel (ILEP) [14] and the joint recommendations of Polish Lipid Association and Polish Society of Laboratory Diagnostics [15]. Therefore, increased awareness of FH is very important.

We aimed to evaluate the clinical features of FH in children and adults based on the data from the PMMHRI Regional Center of Rare Diseases (RCRD) to see the detailed patient’s characteristics, including baseline lipid profile and the time of diagnosis, as well as therapy effectiveness and safety. The novelty of this paper is the common analysis of the population including patients at different ages, which is missing in the background literature. This is also one the first registries describing Polish FH population, and one of few from the Central and Eastern European (CEE) countries.

## 2. Methods

The registry, which is a monocentric retrospective observational study, was conducted in RCRD at the PMMHRI (the second largest, supra-regional hospital in Poland), which is a part of the lipid clinic network of the European Atherosclerosis Society FH-Study Collaboration (EAS-FHSC; https://findmylipidclinic.com). Patients were referred to the clinic from the whole country with the referrals from general practitioners and specialists from other clinics due to dyslipidemia.

All consecutive patients with FH, both children and adults, were enrolled between December 2018 and December 2020. All patients were clearly informed about the study and informed consent was obtained from all of them. All patients received a consultation with a clinical geneticist and a dietitian and were provided with recommendations regarding cascade screening among family members, and a healthy and balanced diet was promoted. All index cases were included in the study. The children included in the study were index cases as well.

The registry is administered by physicians from RCRD of PMMHRI, who solely performed all diagnostic and treatment decisions. The inclusion criteria for the patients with familial hypercholesterolemia were being phenotypically or genetically diagnosed with FH. The registry was established to investigate the clinical characteristics, management, and clinical outcomes data of FH patients. Phenotypic diagnosis was based on the Dutch Lipid Clinic Network criteria (with at least 6 points), which were assessed in all adult patients, and the Simon Broome criteria were assessed in children. Genetic testing was performed with custom panel of 21 genes—the next-generation sequencing (MiniSeq, Illumina, Inc., San Diego, CA, USA), and then the results were analyzed using available databases and predictive programs (sorting intolerant from tolerant (SIFT) and polymorphism phenotyping (PolyPhen)). Confirmation of detected variants was performed with the Sanger sequencing. During the baseline visit, we collected all the data with all the available lipidogram results. We tried to obtain the naïve values of LDL-C, the highest values, and the treated values. All data acquired during the enrollment period were included in the study. Treatment goals were evaluated at the baseline visit, and the decrease of LDL-C and TC was calculated from the naïve values. Data regarding clinical characteristics, such as age, sex, weight, height, body mass index, blood pressure and heart rate, comorbidities, and symptoms, as well as pharmacotherapy, were collected. All source data were revised by investigators. We collected data regarding family history of ASCVD (including coronary syndromes, stroke, transient ischemic attack (TIA), peripheral arterial disease (PAD), and coronary or other arterial revascularization, with presumed atherosclerotic origin). PAD was defined as all arterial diseases excluding aortic and coronary artery diseases [16]. Data on the side effects of statin therapy were collected based on detailed medical history.

### Statistical Analysis

The Shapiro–Wilk test was performed to check normal distribution. Continuous variables were presented as mean ± standard deviation or median and interquartile range when appropriate, and as proportions in the case of categorical variables. Comparisons between groups were performed using the Student’s *t*-test for independent variables, and various nonparametric tests—the Mann–Whitney U test, χ^2^ test with Yates correction, and Kruskal–Wallis test—as appropriate. For all calculations, *p* values < 0.05 were considered statistically significant. Statistical analysis was performed using the STATISTICA v.13 software (TIBCO Software Inc., Palo Alto, CA, USA).

## 3. Results

Of 103 patients with FH, there were 16 children (15.5%) aged 9 ± 3 years and 87 adults (84.5%) aged 41 ± 16 years; 59% were female. The diagnosis of FH in adults was late, at the mean age of 41 years, while in children it was 9 years. Among children and adults with FH, the highest ever recorded levels of total cholesterol (TC) and LDL-C were similar (Table 1). However, children presented with higher mean levels of total cholesterol, LDL-C, and high-density lipoprotein cholesterol (HDL-C) measured at the baseline visit (Table 1). Most of the adults were slightly overweight, and most of the children presented with obesity, which is rather unexpected in a ‘normal’ (non-FH) population. The detailed characteristics of the FH patients are presented in Table 1. Among adults, chronic coronary syndrome (CCS) was diagnosed in 8% patients (n = 7), from which 5.7% (n = 5) of patients had a history of myocardial infarction (MI) and 6.9% (n = 6) of patients had received coronary revascularization. PAD occurred in 2.3% of patients (n = 2) and cerebrovascular disease had occurred in 2.3% of patients (n = 2). Additionally, 62% of adult patients were diagnosed as definite FH according to DLCN criteria and 38% as probable FH. Among children, 68% of patients were classified as definite FH according to Simon Broome criteria and 32% as possible FH.

**Figure 1:**
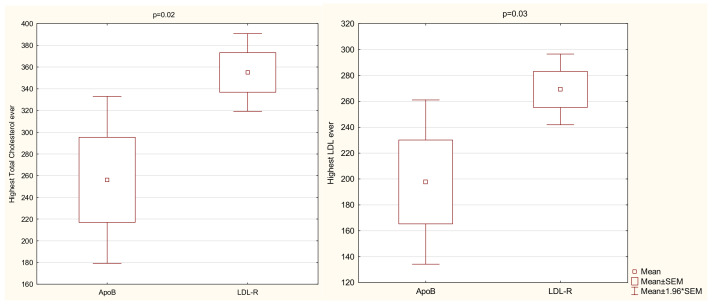
Highest TC and LDL-C ever depending on the underlying gene mutation. Abbreviations: LDL-C—low-density lipoprotein cholesterol, LDLR—low-density lipoprotein receptor, ApoB—apolipoprotein B, Min—minimum, Max—maximum, SEM—standard error of the mean.

**Figure 2:**
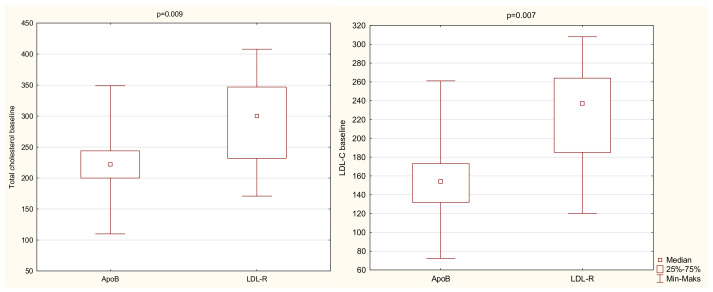
Baseline TC and LDL-C ever depending on the underlying gene mutation. Abbreviations: LDL-C—low-density lipoprotein cholesterol, LDLR—low-density lipoprotein receptor, ApoB—apolipoprotein B, Min—minimum, Max—maximum.

Of 41 patients with available results of genetic testing, there were 29 patients (71%) with a LDLR mutation, 10 patients (24%) with an APOB mutation, and 2 patients (5%) with mutations for whom we did not have the medical history with appropriate data (patients had other documents that confirmed that they had pathological mutations, but with no information regarding the type of mutation). Two patients with a LDLR mutation also presented with concomitant mutations in the APOB gene; one patient had the PCSK9 gene mutation. No one presented with the homozygous form of FH. We observed significant differences in LDL-C and TC measurements at the baseline visit depending on the underlying gene mutation (Figure 1 and Figure 2). Table 2 displays the results of Kruskal–Wallis test clarified by group pairwise comparison presented in Table 3. LDL-C and TC were significantly higher in patients presenting with LDLR mutation rather than APOB mutation. No significant differences were found in the lipidogram between different DLCN categories.

Rates of hypertension and diabetes were relatively low in adult patients (17% and 11%, respectively). In 17% (n = 15) of patients we observed hypertriglyceridemia defined as TG ≥ 150 mg/dL (1.7 mmol/L); most of them presented with mild hypertriglyceridemia, one patient had moderate elevation of TG (≥500 mg/dL (5.64 mmol/L)). Among adult patients, two declared that they were current smokers, while six were previous smokers. The prevalence of chronic coronary syndrome, peripheral artery disease, and stroke in family history in the whole population studied was high (in 44 patients—50%, 17 patients—20%, and 22 patients—25%, respectively).

Most of the adult patients (70%) were prescribed statins (rosuvastatin 70% (n = 42) or atorvastatin 27% (n = 16)) at the baseline visit. The mean doses of used statins were 19 ± 9 mg for atorvastatin and 21 ± 11 mg for rosuvastatin. Furthermore, 14 patients (13.6%) declared statin intolerance, and most of them complained of muscle pain; the rest (4.6%) reported hepatic disturbances. Ezetimibe, as a part of combination therapy, was taken by 29% (n = 25) of adult patients. PCSK9 inhibitors were prescribed for 11.5% (n = 10) within the terms of the drug reimbursement scheme, which required the cumulative fulfillment of the following criteria: age 18 or above; diagnosis of FH with more than 8 points on the DLCN scale; meeting the eligibility criteria for LDL apheresis treatment; and for patients already treated, meeting these criteria at the time of initiation of LDL apheresis treatment. The qualification criteria for LDL apheresis included LDL-C > 160 mg/dL (4.1 mmol/dL) despite diet and either of the following: (a) intensive treatment with statins at maximum doses, i.e., atorvastatin 80 mg or rosuvastatin 40 mg, followed by atorvastatin 40–80 mg or rosuvastatin 20–40 mg in combination with ezetimibe 10 mg, used for a total of 6 months, including combination therapy for a minimum one month; OR (b) intensive treatment with statins at maximum tolerated doses, followed by concomitant treatment with ezetimibe 10 mg, used for a total of 6 months, including combination therapy for a minimum of 1 month [17]. Fibrates were prescribed in 13.7% of adult patients (n = 12) and n-3 fatty acids in 13.7% (n = 12). The frequency of statin use in children was lower than in adults—56% (n = 9) of children were prescribed statins and 19% (n = 3) declared side effects of statin therapy with muscle pain as the side effect of treatment.

Only 50% of adult patients achieved the treatment goal. Depending on the therapy, the highest efficacy of treatment was obtained in patients on the combination triple lipid lowering therapy (statins, ezetimibe, and PCSK9 inhibitors; n = 9). The mean LDL cholesterol in that group of patients was 59 ± 33 mg/dL. In patients treated with dual therapy (statins and ezetimibe), the effectiveness of treatment was lower. The effectiveness of the pharmacotherapy is presented in Table 4. In children treated with statins, the mean decrease of total cholesterol was 41% (mean absolute reduction by 147 ± 42 mg/dL (3.8 ± 1.09 mmol/L)) and for LDL cholesterol it was 44% (mean absolute reduction by 122 ± 47 mg/dL (3.15 ± 1.22 mmol/L)). None of children achieved the treatment goal.

Adult patients who achieved the treatment goal had a higher DLCN score (mean: 17; SD: 16–19 vs. 6.5; 6–10, *p* = 0.003) and glucose measured at baseline (103; 96–112 vs. 91; 86–96, *p* = 0.03) in comparison to patients who did not achieve the treatment goal. Other factors that correlated with the ability to achieve the treatment goal were a premature history of coronary artery disease (*p* = 0.002), history of myocardial infarction (*p* = 0.02), history of revascularization (*p* = 0.04), treatment with angiotensin-converting enzyme inhibitors (ACE-I) (*p* = 0.02), and treatment with ezetimibe (*p* = 0.01) and with PCSK9 inhibitors (*p* = 0.002). Treatment with statins (both statin monotherapy or any statin therapy as part of a lipid-lowering regimen) was not linked with the ability to reach treatment goals (*p* = 0.15).

## 4. Discussion

We report the preliminary results of the registry of children and adults with FH conducted in PMMHRI. We showed that only 50% of adult patients and none in the group of children were able to achieve the LDL-C target, which was also associated with the lipid-lowering therapy under-prescription and unsuitable doses of statins administrated in the pre-referral period. Based on our analysis, the higher risk of the patients at baseline, the higher chance to be on LDL-C target, especially those being treated with the combination dual and triple therapy, which is still relatively rare. Statin monotherapy does not increase the chances of achieving the goal. Effective treatment of children is still a large challenge, even in specialist centers.

Besides optimal pharmacotherapy, an introduction of healthy diet is highly advised for each patient with FH. At this point, it should be emphasized that we observed that while adults with FH were mostly of normal weight or overweight, and the majority of children were obese. Therefore, the implementation of healthy diet should be initiated as early as possible [18]. In our population, all the patients were advised about how to implement healthy lifestyle including a well-balanced diet. It seems that the help of certified nutritionist/dietitian and the involvement of the whole family is crucial to achieve optimal dietary habits. As part of a balanced diet for FH, patients should consider the inclusion of functional foods such as plant stanols and sterols, which lower LDL cholesterol [19]. Physical activity is also very important. Before implementation of regular exercise to a patient’s daily routine, cardiovascular function should be assessed, especially for adult patients with FH [13,20]. Moreover, all patients should be warned about potential muscle aches and pains to avoid minor skeletal injuries resulting from exercise being misattributed to statin therapy [21].

Despite increased social awareness of the importance of treating hyperlipidemia, FH is still highly underdiagnosed and undertreated and remains a significant challenge to public policy. We confirmed it in our analysis showing that the age at which FH was diagnosed was relatively high (however, younger in comparison to the other analyses from this region [22]) both in adults (mean age of 41 years) and in children (9 years), what is also raised in all available registries and data from different countries [20]. It is recognized that, if left untreated, FH patients develop ASCVD before the age of 55 in men and 60 in women [23].

The level of highest TC and highest LDL-C observed in our population was similar to the values observed in other published data [20,24]. The mean highest recorded TC ever for the individuals in our population was 335 ± 85 mg/dL (8.66 ± 2.2 mmol/L), while available data show that this is between 310 and 580 mg/dL (8.02–15 mmol/L) for untreated patients [20,24].

The prevalence of ASCVD in our population was relatively low (which might have been due to the relatively young age of FH diagnosis—41 years); we observed chronic coronary syndrome in 11.5% patients, from which 5.7% patients had a history of myocardial infarction and 7% patients required revascularization and peripheral artery disease, and cerebrovascular disease was reported in 3% patients. However, the clinical manifestation of FH usually starts at fourth decade of life [24], which may also explain the relatively low prevalence of ASCD in our population. However, it is worth emphasizing that the prognosis of these patients is poor, as the concept of lifetime LDL exposure dictates that an untreated FH patient aged 45 years has a similar lifetime exposure to LDL-C as a 70-year-old without FH [25].

Therefore, pharmacological treatment should be initiated as early as possible in FH. The recommended LDL-C target for adults with FH at very high CVD risk is <55 mg/dL (<1.4 mmol/L) and a reduction of >50% from baseline, both in primary and secondary prevention, and for patients with FH at high risk, an LDL reduction of >50% from baseline and a goal of 70 mg/dL (1.8 mmol/L) is recommended (however, it is important to notice that these are rare cases of such patients with FH and no other risk factors, and/or without subclinical atherosclerosis) [6,13]. However, based on data from available trials [26,27,28], there are additional suggestions available in the literature both for risk stratification and for the recommended LDL-C targets. These proposals emphasize the fact that patients with FH (those with concomitant ASCVD/acute coronary syndrome) should be considered as having an extremely high CVD risk because of a high cumulative burden of LDL-C. Therefore, a lower LDL-C target (<40 mg/dL (<1 mmol/L)) is warranted [15,29].

The treating of FH children patients is a great challenge and unmet need. Unfortunately, there are still no clear guidelines about treatment in children. However, the most important issue seems to be an early initiation of therapy to minimize the cumulative burden of LDL-C [18]. The available data suggest that the therapy with statins may be initiated at the age of 6 years, while ezetimibe may be started at the age of 10 years. Other treatment options include bile acid sequestrants [30,31]. In our population, none of the children achieved the treatment goal, which could be a result of overcautious under-dosing in children.

In adults with FH and concomitant ASCVD, we should start with dual or triple lipid-lowering therapy [15,29], bearing in mind the fact that for most patients at very high or extremely high risk of cardiovascular events, the target can only be achieved with combination therapy [29,32]. In addition to statins and ezetimibe, proprotein convertase subtilisin/kexin type 9 inhibitors (PCSK9 inhibitors) increase the likelihood of achieving treatment targets [29,32]. In our population, the percentage of adults receiving triple therapy was low and was limited to patients who had reimbursement by the National Health Fund of Poland [17]. These findings highlight the poor availability of PCSK9 inhibitors, especially the mean reduction of TC and LDL-C in patients treated with combined triple therapy was highest and allowed for the achievement of LDL-C targets in most of the patients. The percentage decrease of LDL-C observed in our population is comparable to the observations made by other researchers [33,34,35,36]. The ODYSSEY long-term study evaluated alirocumab vs. placebo in 2341 patients with HeFH with or without ASCVD or with its risk equivalents. PCSK9 inhibitors, added to lipid-lowering treatment in patients who were not adequately controlled, reduced LDL-C by 61.0% vs. 0.8% change with placebo (CI: −64.3 to −59.4; *p* < 0.001) [31]. The ODYSSEY ALTERNATIVE study that evaluated 314 patients with HeFH with a high CV risk and a history of statin intolerance showed that alirocumab reduced LDL-C by up to 45% (vs. 14.6% with ezetimibe) after 24 weeks of treatment (*p* < 0.0001) [32]. Another two studies—ODYSSEY FH I and ODYSSEY FH II—showed a reduction of LDL by 48.8% for alirocumab compared with an increase of 9.1% for placebo (*p* < 0.0001) and a 48.7% reduction compared with a 2.8% increase with placebo (*p* < 0.0001), respectively [33]. A slightly smaller decrease in LDL-C was observed among 105 patients included in the ODYSSEY HIGH FH trial. Alirocumab has been shown to reduce LDL cholesterol by 46% (vs. 7% for placebo with *p* < 0.001) [36]. The population enrolled in this study is very similar to a group of patients from our registry treated with triple therapy (based on the reimbursement criteria), because inclusion criteria assumed the maximally tolerated dose of statins and LDL cholesterol higher than 160 mg/dL. Despite an absolute mean LDL cholesterol reduction, the goal of therapy was achieved only in 41% of individuals, which is consistent with the results of our study [36].

In the literature, there are other recently published registries of different populations including the following: Iran Registry of FH (IRFH) [37], Arabian Gulf Region Registry—the Gulf FH Registry [38], Taiwanese Registry [39], Mexico Registry [40], Italian Atherosclerosis Society Network Registry (LIPIGEN) [41], and Spanish Familial Hypercholesterolemia Longitudinal Study (SAFEHEART) [42]. All those registries are national ones with higher number of included patients. The IRFH included 263 adult patients with probable or definite FH with lower than in our population LDL cholesterol levels (142.42 ± 45.27 mg/dL, 3.68 ± 1.17 mmol/L) and lower number of patients on LLT treatment (54.9%) [37]. The Gulf FH registry, which was multicenter and multinational study, included 306 adult patients with probable or definite FH. This registry showed a low rate of treated patients, and statin therapy was present in 34%, ezetimibe in 10%, and PCSK9 inhibitors in 0.4% of patients. The treatment goals regarding the LDL cholesterol level were achieved only in 12% of patients [38]. The Mexican FH Registry included 336 adult patients. Genetic testing was performed in 26.9% of patients. The authors observed higher prevalence of statin therapy (88.1%) than in our population. Ezetimibe was added to LLT in 35.7% of patients. However, the treatment goal was achieved only in 13.7% of patients [40]. All those registries conclude that still most of FH patients are underdiagnosed and undertreated [37,38,40]. The biggest Taiwanese registry was aimed to assess the spectrum of genetic mutations in adult patients with clinically diagnosed FH. Similar to our population, most of the patients presented with a LDLR gene mutation (89%). The APOB gene mutation was present in 13% of patients. The PCSK9 mutation was not found in the population studied. The authors identified four novel mutations [39]. It should be emphasized that our registry included both children and adults, which broadens our knowledge about the characteristics of FH in all patients. On the other hand, the interpretation of the obtained results may be more difficult. The Italian registry LIPIGEN, which is an observational, multicenter retrospective and prospective study, included 3480 patients with DLCN score ≥6, among whom 15% were under 18 years. The authors reported a higher prevalence than in our population of coronary artery disease (11.3%), peripheral vascular disease, stroke (9.6%), and family history of coronary artery disease (38.8%) [41]. Another registry, which included children was the SAFEHEART registry, an open, multicenter, Spanish prospective study, which included patients with FH and their relatives older than 15 years. Despite LLT (84% patients received LLT), most of studied population had high levels of LDL-C, not reaching therapeutic goal. Only 3.4% of patients reached LDL-C < 100 mg/dL (2.59 mmol/L) [42].

However, despite many reports already available concerning FH patients in the literature, we believe that the knowledge on FH is still lacking. In most of the countries worldwide, the number of patients diagnosed is usually less than 1–3%. Therefore, each new report is of great importance. It is especially true for CEE countries, including Poland, and based on our best knowledge it is only the second real-life report on FH population [43] and one of several from this region [22,44]. Moreover, there are large differences in the diagnosis, effectiveness of therapy, and monitoring of FH patients in different regions of the world, and such papers give possibility to compare these outcomes. Finally, this is one of the few reports that jointly evaluates children and adults with FH, showing some large differences and unmet needs concerning the diagnosis and therapy.

Our analysis has some obvious limitations. This is an analysis of a selected patient population with FH. The main limitation of the study is limited size of the study groups, due to its preliminary report character; however, the registry is ongoing, and these results will be validated in the further analyses. However, despite the small cohort size, the study remains informative. The study group consists of both adults and children, which makes the group heterogeneous but, on the other hand, gives a more complex picture of the FH patient population. Another limitation is some form of selection bias that perhaps the most serious cases are more likely to be diagnosed in childhood, whereas the less serious cases might escape detection until adulthood.

## 5. Conclusions

In conclusion, despite the younger age of FH diagnosis, children presented with higher mean levels of LDL cholesterol than adults. Therefore, the lifetime exposure to LDL cholesterol starts at a very young age. As a result, there is a need to enable the earlier initiation of therapy and the strict monitoring of the progression of atherosclerosis. Late diagnosis of FH in adults, as well as therapy under-prescription and ineffectiveness also represent an unmet clinical need and is likely to be associated with a poorer prognosis.

## Figures and Tables

**Table 1:** Clinical characteristics and laboratory tests in patients with FH.

	Children (n = 16)	Adults (n = 87)	*p*	Total
Clinical characteristics				
Age at baseline visit (years)	9 ± 3	41 ± 16	<0.01	39 ± 18
Weight (kg)	37 ± 17	82 ± 17	<0.01	73 ± 25
Height (m)	146 (124–159)	170 (163–176)	<0.01	165 (160–175)
Body mass index (kg/m^2^)	25.3 ± 1.8	28.4 ± 5.3	0.42	28.2 ± 5.2
Systolic blood pressure (mmHg)	112 (104–116)	135 (120–150)	0.001	128 (117–147)
Diastolic blood pressure (mmHg)	68 (65–74)	82 (73–92)	0.004	77 (71–90)
Heart rate (bpm)	70 (60–71)	75 (63–80)	0.16	71 (60–80)
Laboratory test results				
Highest TC ever (mg/dL)	343 ± 61	333 ± 91	0.74	335 ± 85
Highest LDL ever (mg/dL)	266 ± 51	235 ± 69	0.18	241 ± 66
TC (mg/dL)	313 (279–354)	259 (213–334)	0.04	266 (220–336)
LDL (mg/dL)	247 (206–260)	192 (144–251)	0.02	195 (151–257)
HDL (mg/dL)	53 (46–67)	48 (40–58)	0.009	48 (40–59)
TG (mg/dL)	84 (67–101)	112 (83–175)	0.15	107 (79–162)
Glucose (mg/dL)	83 (82–88)	93 (86–97)	0.04	90.5 (82–96)
AspAT (U/L)	24 (22–29)	24 (19–29)	0.69	24 (19–29)
AlAT (U/L)	19 (15–27)	23 (17–33)	0.21	22 (15–33)
Creatinine kinase (U/L)	114 (58–149	76 (63–133)	0.54	86 (62–140)
TSH (µLU/mL)	1.72 (1.43–2.1)	1.77 (1.38–2.57)	0.89	1.73 (1.43–2.46)
Pharmacotherapy				
Statins (%)	56	70		
Ezetymibe (%)	0	29		
PCSK9 inhibitors (%)	0	11.5		
Fibrates (%)	0	13.7		
n-3 fatty acids (%)	0	13.7		

Abbreviations: TC—total cholesterol, bpm—beats per minute, LDL—low-density lipoprotein, HDL—high-density lipoprotein, TG—triglycerides, AspAT—aspartate transaminase, AlAT—alanine transaminase, TSH—thyroid-stimulating hormone, PCSK9—proprotein convertase subtilisin kexin type 9.

**Table 2:** An association between mutation type and lipidogram.

Paired Differences									
	Mean	SD	Median	Interquartile Range	Min	Max	X^2^	df	*p*
Highest TC ever	335	85	329	282–390	142	600	0.7	3	0.13
Highest LDL-C ever	241	67	243	196–293	80	400	3.5	2	0.19
TC at baseline	274	71	267	220–336	110	419	8.5	2	0.02
LDL-C at baseline	200	64	195	151–257	55	331	3.9	2	0.02
HDL-C at baseline	51	15	48	40–59	27	102	2.5	2	0.81
TG at baseline	136	112	107	79–162	33	842	6.3	2	0.09

Abbreviations: SD—standard deviation, Min—minimum, max—maximum, X2—chi-square, df—degrees of freedom, TC—total cholesterol, LDL-C—low-density lipoprotein cholesterol, HDL-C—high-density lipoprotein cholesterol, TG—triglycerides.

**Table 3:** Post hoc pairwise comparison for independent Kruskal–Wallis (KW).

	Pairwise Comparison—Gene mutation	Mean Ranks	*p* of KW
LDL-C at baseline	Lack—LDLR	21.0/20.3	1.0
	LDLR—ApoB	20.3/9.7	0.02
	Lack—ApoB	21.0/9.7	0.44
TC at baseline	Lack—LDLR	26.0/19.8	1.0
	LDLR—ApoB	19.8/9.7	0.03
	Lack—ApoB	26.0/9.7	0.11
TG at baseline	Lack—LDLR	28.5/16.1	0.19
	LDLR—ApoB	16.1/12.7	1.0
	Lack—ApoB	28./12.7	0.08

Abbreviations: KW—Kruskal–Wallis, LDL-C—low-density lipoprotein cholesterol, TC—total cholesterol, TG—triglycerides, LDLR—low-density lipoprotein receptor, ApoB—apolipoprotein B.

**Table 4:** Effectiveness of pharmacotherapy.

	TC	LDL-C
	Triple lipid lowering therapy	
Mean decrease (%)	58	71
Mean absolute reduction	200 ± 121 mg/dL (5.17 ± 3.13 mmol/L)	190 ± 92 mg/dL (4.91 ± 2.38 mmol/L)
	Dual therapy	
Mean decrease (%)	41	38
Mean absolute reduction	174 ± 59 mg/dL (4.5 ± 1.53 mmol/L)	108 ± 75 mg/dL (2.79 ± 1.94 mmol/L)
	Statins	
Mean decrease (%)	38	45
Mean absolute reduction	139 ± 65 mg/dL (3.59 ± 1.68 mmol/L)	119 ± 57 mg/dL (3.08 ± 1.47 mmol/L)
	Ezetimibe	
Mean decrease (%)	4	5
Mean absolute reduction	14 mg/dL (0.36 mmol/L)	14 mg/dL (0.36 mmol/L)

Abbreviations: TC—total cholesterol, LDL-C—low-density lipoprotein cholesterol.
